# 4D flow CMR analysis comparing patients with anatomically shaped aortic sinus prostheses, tube prostheses and healthy subjects introducing the wall shear stress gradient: a case control study

**DOI:** 10.1186/s12968-020-00653-9

**Published:** 2020-08-10

**Authors:** Malte Maria Sieren, Victoria Schultz, Buntaro Fujita, Franz Wegner, Markus Huellebrand, Michael Scharfschwerdt, Hans-Hinrich Sievers, Joerg Barkhausen, Alex Frydrychowicz, Thekla Helene Oechtering

**Affiliations:** 1 Department for Radiology and Nuclear Medicine, Ratzeburger Allee 160, 23562 Lübeck, Germany; 2 Department for Cardiac and Cardiothoracic Vascular Surgery, University Hospital Schleswig-Holstein, Lübeck, Germany; 3 Fraunhofer Institute for Digital Medicine MEVIS, Bremen, Germany

**Keywords:** 4D flow MRI, Wall shear stress, Valve-sparing aortic root replacement, VSARR, Thoracic aortic aneurysm, Aortic tube prosthesis, Aortic sinus prosthesis, Secondary flow pattern, Hemodynamics, Post-operative outcome

## Abstract

**Background:**

Anatomically pre-shaped sinus prostheses (SP) were developed to mimic the aortic sinus with the goal to preserve near physiological hemodynamic conditions after valve-sparing aortic root replacement. Although SP have shown more physiological flow patterns, a comparison to straight tube prosthesis and the analysis of derived quantitative parameters is lacking. Hence, this study sought to analyze differences in aortic wall shear stress (WSS) between anatomically pre-shaped SP, conventional straight tube prostheses (TP), and age-matched healthy subjects) using time-resolved 3-dimensional flow cardiovascular magnetic resonance (4D Flow CMR). Moreover, the WSS gradient was introduced and analyzed regarding its sensitivity to detect changes in hemodynamics and its dependency on the expression of secondary flow patterns.

**Methods:**

Twelve patients with SP (12 male, 62 ± 9yr), eight patients with TP (6 male, 59 ± 9yr), and twelve healthy subjects (2 male, 55 ± 6yr) were examined at 3 T with a 4D Flow CMR sequence in this case control study. Six analysis planes were placed in the thoracic aorta at reproducible landmarks. The following WSS parameters were recorded: WSS_avg_ (spatially averaged over the contour at peak systole), max. WSS_seg_ (maximum segmental WSS), min. WSS_seg_ (minimum segmental WSS) and the WSS Gradient, calculated as max. WSS_seg_ – min. WSS_seg_. Kruskal-Wallis- and Mann-Whitney-U-Test were used for statistical comparison of groups. Occurrence and expression of secondary flow patterns were evaluated and correlated to WSS values using Spearman’s correlation coefficient.

**Results:**

In the planes bordering the prosthesis all WSS values were significantly lower in the SP compared to the TP, approaching the physiological optimum of the healthy subjects. The WSS gradient showed significantly different values in the four proximally localized contours when comparing both prostheses with healthy subjects. Strong correlations between an elevated WSS gradient and secondary flow patterns were found in the ascending aorta and the aortic arch.

**Conclusion:**

Overall, the SP has a positive impact on WSS, most pronounced at the site and adjacent to the prosthesis. The WSS gradient differed most obviously and the correlation of the WSS gradient with the occurrence of secondary flow patterns provides further evidence for linking disturbed flow, which was markedly increased in patients compared to healthy sub jects, to degenerative remodeling of the vascular wall.

## Background

The established therapy for aneurysms of the aortic bulb and ascending aorta is the implantation of a tubular prosthesis while preserving the aortic valve (valve sparing aortic root replacement, VSARR). Conventional tube prostheses (TP) are known to interfere with the opening of the aortic valve, inducing flow diversions in the aorta and changes in the vessel’s anatomy like kinking, post-prosthetic dilatation, and increased transvalvular pressure differences [1–4]. Consequently, anatomically pre-shaped sinus prostheses (SP) were developed to mimic the aortic sinus with the goal to preserve near physiological hemodynamic conditions [2, 5–8]. Yet, the clinical significance of this innovation remains a subject of controversy [9].

Time-resolved 3-dimensional cardiovascular magnetic resonance (CMR) phase contrast imaging (4D Flow CMR) has provided unprecedented insights into hemodynamics of the aorta in vivo, offering visualization of macroscopic blood flow behavior as well as quantification of flow- and velocity-derived parameters [10]. There are several works confirming altered aortic hemodynamics after conventional VSARR [11–14]. Of note, in a previous study using 4D Flow CMR, we could confirm almost physiological flow patterns in the aortic root of the SP compared to disturbed flow in the TP [15]. Nonetheless, distal to both prostheses types, significantly more secondary flow patterns than in healthy subjects were detected [8, 16]. These secondary flow patterns were only evaluated semi-quantitatively on a Likert scale; the study lacked a quantitative parameter to objectively quantify flow abnormalities. While secondary flow patterns are often used as markers for the presence of pathological hemodynamics or even for a specific pathology, e.g. pulmonary artery hypertension [17, 18], the effect of these secondary flow patterns on pathological changes of the vessel wall architecture and thus the long-term morbidity of patients exhibiting these flow patterns remain a subject of debate.

We hypothesize that an increase in flow disturbances results in pathological, elevated mechanical stress to the vessel wall. A parameter able to quantify these changes is the wall shear stress (WSS). WSS is a force by blood flow acting tangentially through mechano-transduction on the endothelial cells of the vessel wall. Altered values are linked to pathological vessel wall changes, including remodeling of the vessel wall, atherosclerotic plaque induction, and potential aneurysm growth [19–22]. Recent studies evaluating WSS concentrate on averaged (WSS_avg_) and maximum segmented WSS (WSS_seg_) values thereby neglecting minimal values. As both, WSS maxima and minima do affect the vessel architecture [23, 24], a comprehensive parameter depicting the gradient between maximum and minimum WSS values potentially provides valuable information to assess hemodynamics and its effects on the vessel wall.

So far, there is only one study assessing WSS in patients who received prostheses with neo-sinus with a relative small number of examined patients [16]. As anatomically pre-shaped SP are specifically designed to re-establish near physiological flow conditions, a comprehensive analysis investigating the interdependencies of pathological flow patterns on WSS and other quantitative flow parameters in SP compared to conventional TP is of fundamental interest, potentially affecting post-procedural patient morbidity and surgical strategies in the long term.

Hence, the aim of this work was to compare quantitative parameters derived from 4D Flow CMR with focus on the newly introduced WSS gradient in patients after implantation of SP to patients who received conventional TP and to age-matched, healthy subjects. In a sub-analysis, the dependency of WSS on the expression of secondary flow patterns was investigated.

## Methods

### Study participants

The study population consisted of three cohorts: Twelve patients with SP, (12 male, 62 ± 9yr; SP, Uni-Graft W SINUS; Braun, Melsungen, Germany), eight patients with conventional, TP (6 male, 59 ± 9yr), and twelve age-matched, healthy subjects (2 male, 55 ± 6yr). Table 1 provides a comprehensive demographic description of study participants. All subjects were enrolled in this HIPAA-compliant study after approval of the local ethics committee and written informed consent. They were consecutively recruited through the Department for Cardiac Surgery outpatient service and underwent routine follow-up CMR including an additional 4D Flow CMR scan. Patients were recruited dependent on the implanted graft type: patients with TP were scanned and added to a previously presented study collective focused on the semi-quantitative evaluation secondary flow patterns [8]. Previous data were re-evaluated for the purpose of the presented hypotheses.

**Table 1:** Demographics and clinical data of the studies participants

	Pat SP		Pat TP		VOL		p	p		
	Mean ± SD	range	Mean ± SD	range	Mean ± SD	range	all	SP vs. TP	Vol vs. SP	Vol vs. TP
Age [years]	55 ± 15	(26–73)	61 ± 13	(53–72)	55 ± 6	(47–69)	**0.03**	**0.04**	0.42	**0.04**
Weight [kg]	89 ± 9	(76–100)	83 ± 15	(78–92)	69 ± 13	(53–92)	0.53	0.81	**< 0.01**	0.10
Gender ratio [male:female]	11:1		8:1		2:10					
Height [cm]	183 ± 10	(160–200)	178 ± 6	(174–182)	171 ± 7	(161–183)	0.10	0.33	**< 0.01**	0.08
BMI [kg/m2]	27 ± 4	(21–32)	25 ± 6	(23–28)	24 ± 3	(20–30)	0.44	0.98	0.30	0.38
Blood pressure [mmHg]	147 ± 21/81 ± 9	115–180/60–95	135 ± 12/85 ± 7	(125–146/80–89)	127 ± 19/80 ± 10	110–160/70–100)	0.04/0.08	0.24/0.27	**0.03**/0.50	**0.02/0.03**
Aortic valve, [tri−/bicuspid]	9/3		8/0		12/0					
Heart rate [bpm]	68 ± 11	(52–87)	61 ± 6	(56–63)	63 ± 9	(49–76)	0.22	0.50	0.88	**0.02**
Ejection fraction [%]	58 ± 7	(43–66)	51 ± 12	(49–58)	65 ± 3	(61–69)	0.14	0.85	**0.02**	0.08

Significant differences are marked in bold type. *SP* Sinus prosthesis, *TP* tube prosthesis, *BMI* body mass index, *bpm* beats per minute

### CMR scans

CMR was performed on a 3 T scanner (Philips Ingenia Omega dStream, R5.18, Philips Healthcare, Best, The Netherlands) using a 20-channel body surface coil. For 4D Flow CMR acquisitions, a retrospectively electrocardiogram (ECG)-gated, time-resolved, three-dimensional, cartesian phase-contrast CMR sequence with referenced three-directional velocity-encoding (VENC) was used. Respiratory gating with an acceptance window of 8–12 mm to achieve a gating efficiency on the order of 60% was applied. Typical imaging parameters were VENC = 180–200 cm/s and time to repeat/time to echo 3.6/2.3 ms. Data were acquired with an isotropic resolution of 2.4 mm, reconstructed to 2 mm and 20 time frames per RR-interval. An effective temporal resolution of 31–67 ms was achieved. Parallel imaging with a sensitivity encoding (SENSE) acceleration factor of 2 resulted in scan times to PO provided page range in new reference 8 if fine to proceed. Thank you between 9 and 18 min, depending on the individual heart rate and navigator gating efficiency. Eddy currents and Maxwell terms were corrected automatically during offline reconstruction. All imaging parameters were in concordance with the consensus paper by Dyverfeldt et al. [25]. Contrast agent (1.0 mmol gadobutrol, 0.1 ml/kg BW, Bayer HealthCare, Berlin, Germany) was administered per clinical routine in all patients and 2 healthy subjects, the flip angle was adapted accordingly to 7° in studies without and 14° with contrast agent.

### Quantitative data analysis

Data were transferred to an offline workstation for postprocessing, visualization and quantification. WSS analysis was performed using GTFlow (v3.1.14; GyroTools LLC, Zurich, Switzerland). In six patients, data processing necessitated aliasing correction using PhaseUnwrappingTool (Fraunhofer MEVIS, Bremen, Germany).

**Fig. 1:**
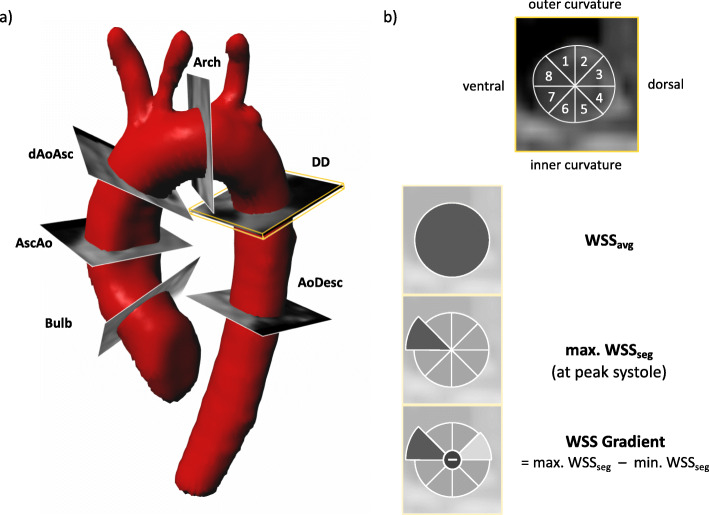
Location of contours in the aorta. **a** Exemplary depiction of contour placement in a 4D Flow dataset of a healthy subject. Contours were placed at reproducible anatomical landmarks at the aortic bulbus (bulbus), halfway between the valve and the origin of the brachiocephalic trunk in the ascending aorta (AoAsc), proximal to the origin of the brachiocephalic trunk in the distal ascending aorta (dAscAo), between the origins of the left common carotid and subclavian artery in the aortic arch (Arch), at the ductus diverticulum (DD) and in the descending aorta (AoDesc). Correct placement was confirmed via a virtual angiography generated from complex difference data. **b** Each contour was divided into 8 equally sized sub-segments for wall shear stress (WSS) analysis. To ensure for spatial comparability between planes segments 1 and 2 were orientated to face the outer curvature of the aorta. Recorded WSS parameters were: WSS averaged per plane (WSS_avg_); maximum WSS per segment (max. WSS_seg_); WSS Gradient (WSS_Grad_), calculated as maximum WSS_seg_ – minimum WSS_seg_

Six analysis planes were placed orthogonal to the vessel’s axis at reproducible anatomical landmarks: the aortic bulb (bulb), halfway between the valve and the origin of the brachiocephalic trunk in the ascending aorta (AAo), proximal to the origin of the brachiocephalic trunk in the distal ascending aorta (dAAo), between the origins of the left common carotid and the left subclavian artery in the aortic arch (AA), at the location of the ductus diverticulum (DD), and at the level of the first plane in the descending aorta (DAo) (Fig. 1). Vessel-contours were manually segmented using a B-spline algorithm and carefully fitted to the vessel’s margins in each time step to account for vessel movement. Correct placement of contours was confirmed via a surface shaded 3D volume angiography based on the velocity-weighted magnitude data (complex difference). Hemodynamic and geometrical parameters stroke volume [ml], maximum flow [ml/s], maximum velocity [cm/s] and maximum area [mm^2^] were reported. For the WSS analysis, each contour was automatically divided into 8 sub-sections, which were manually aligned to the aortic anatomy (outer curvature [sections 1–2], ventral curvature [sections 3–4], inner curvature [sections 5–6], dorsal curvature [7, 8]). The following WSS-parameters were recorded in N/m^2^ units: Temporal maximum WSS at peak systole, averaged per plane/contour (WSS_avg_); temporal maximum segmental WSS at peak systole (max. WSS_seg_); minimum segmental WSS (min. WSS_seg_) at the respective timepoint of max. WSS_seg_;the WSS gradient (WSS_grad_) was calculated as maximum WSS_seg_ – minimum WSS_seg_. Location of contours and systematics of different WSS parameters are illustrated in Fig. 1.

### Analysis of blood flow

Blood flow pattern visualization was achieved via time-resolved pathlines and instantaneous 3D streamlines emitted up- and downstream from each predefined contour. Data were color-coded with respect to the acquired flow velocity.

**Fig. 2:**
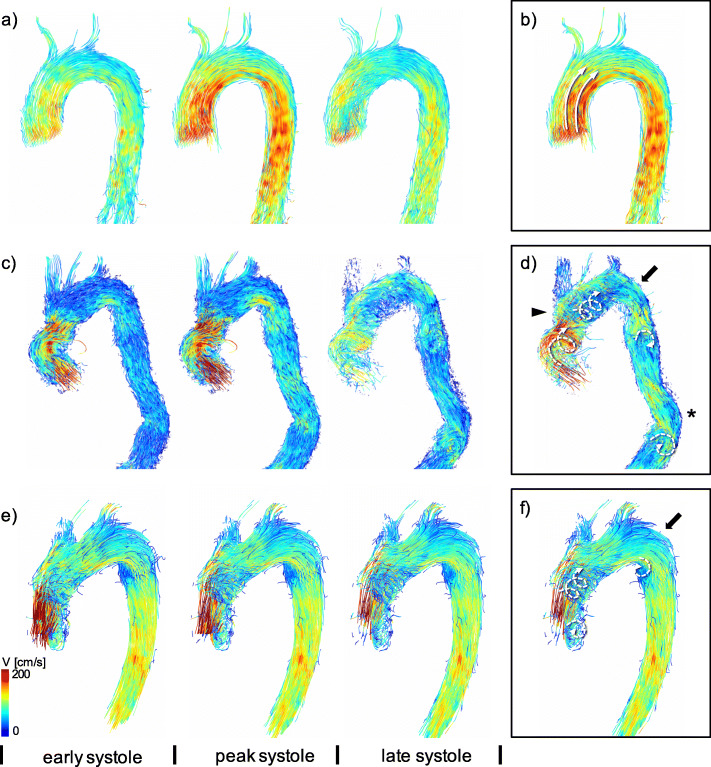
Comparison between two patients after valve-sparing aortic root replacement and a healthy subject. 4D Flow data of **a**) a healthy subject, **c**) a patient with a conventional tube prosthesis (TP) and **e**) a patient with a sinus prosthesis (SP) replacing the aortic bulb and ascending aorta. Blood flow is visualized starting from the ascending aorta using 3D pathlines, color-coded with respect to flow velocity (V [cm/s]). Images depict the generation of the flow field from left to right in early systole, late systole and early diastole. Flow patterns in peak systole are traced in **b**), **d**) and **f**). A variety of secondary flow patterns (dotted arrows) form in the aorta of both patients in **d**) and **f**). The subject in **b**) demonstrates undisturbed physiological flow. Both patients show typical changes in the aortic geometry including post-prosthetic dilatation (triangle), post-prosthetic kinking (bold arrow) and elongation of the descending aorta (asterisk)

Vortices in the sinus and helical flow taking the full diameter of the aortic vessel are examples of physiological, primary flow patterns, which can be found in healthy subjects. Vortices and helices were marked as secondary flow patterns when differing from these physiological primary flow patterns. The presence of secondary vortices was defined as concentric, circular flow in opposition to the main direction of flow within the vessel [26]. A secondary helix was defined as concentric flow in the main flow direction, not filling the complete cross-section of the aorta [27]. Figure 2 depicts exemplary data from a patient study with secondary flow patterns and a healthy subject scan with physiological, undisturbed flow.

**Fig. 3:**
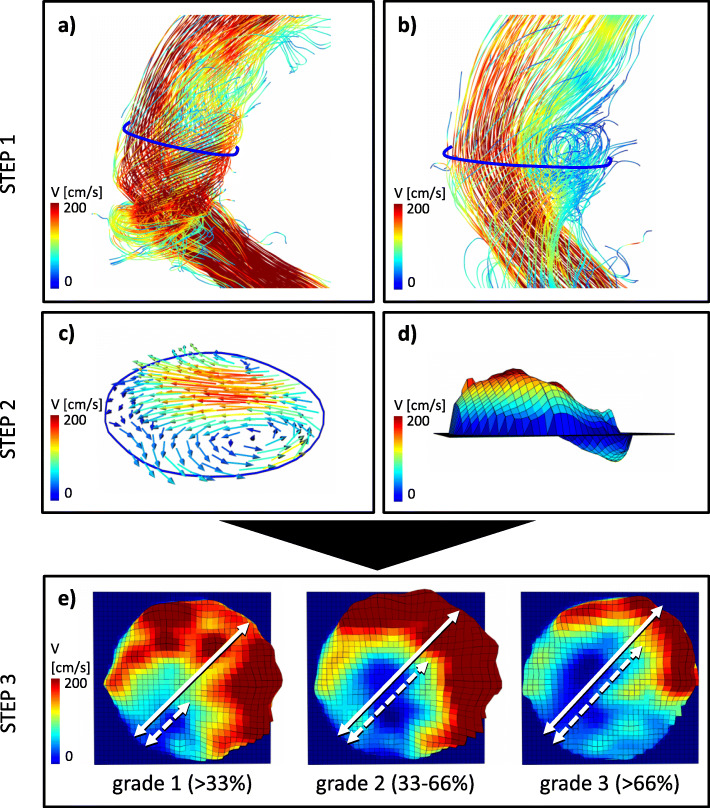
Grading scheme for secondary flow patterns. In a first step, the flow field was generated using pathlines and 3D streamlines, color-coded with respect to flow velocity (V [cm/s]), to detect secondary flow patterns. **a** demonstrates a secondary helix, **b** a secondary vortex in the ascending aorta. Secondly, after a secondary flow pattern was detected, a scalar map and in plane vectors were created to confirm the respective characteristics of **c** a helix: helical flow, no backward flow, and **d** a vortex: helical flow optional, backward flow. **e** Lastly, the secondary flow pattern was graded on a scale of 1–3 with respect to the proportion of aberrant flow (dotted arrow) to the aorta’s diameter (bold arrow). The grading scheme is based on the method by Oechtering and coworkers [8]

Flow patterns were analyzed if they involved one of to the previously defined contours. After detection of a secondary flow pattern in the macroscopic 3D flow field, a scalar map and in plane vectors were created within the adjacent contour to distinguish between helix (helical flow, no backward flow) and vortex (helical flow optional, backward flow). In the next step, distribution of aberrant flow in the vessel’s area was evaluated on a scalar map. Secondary flow patterns were graded in semi-quantitative fashion on a scale of 0–3 as shown before with respect to the diameter of aberrant flow in proportion to the aorta’s diameter [15]. The presence and grading of each secondary flow pattern was reported for each contour accordingly. The grading scheme of secondary flow patterns is illustrated in Fig. 3.

### Statistics

Statistical analyses were performed using SPSS® (Version 25.0. Statistical Package for the Social Sciences, International Business Machines, Inc., Armonk, New York, USA). After testing for normal distribution applying Shapiro-Wilk test values are presented as average ± standard deviation. To minimize error due to multiple testing differences for WSS, hemodynamic and geometrical parameters between all groups were tested first using Kruskal-Wallis-Test. Subsequently, each patient group (SP and TP) was compared to each other and individually to healthy, aged matched subjects in pairs by means of Man-Whitney-U-Test.

Irrespective of the study participant’s status as patient/volunteer, peak WSS values and the WSS gradient were correlated with the presence and grading of secondary flow patterns per contour by applying Spearman’s correlation coefficient. Box plots where calculated to visualize distribution of WSS values depending on the grading of secondary flow patterns.

For the inter-reader comparison, a second observer reevaluated a subset of six individuals, two randomly selected from each group. For WSS values, the intraclass correlation coefficient (ICC; two-way mixed model; absolute agreement; single measurement) with 95% confidence intervals was computed. For inter-reader comparison of flow pattern grading Cohen’s kappa coefficient was used.

## Results

### Quantitative parameters

Table 2 summarizes the results of the WSS analysis. The WSS gradient was the best parameter to discriminate between groups and showed significantly different values in the ascending aorta and the aortic arch when comparing both prostheses to healthy subjects. In the DAo, only the difference between healthy subjects and patients with TP were statistically significant. Comparing both prostheses against each other, there was a significant difference for WSS gradient in the bulbus and in the ascending aorta. Although values were still elevated in the SP compared to healthy subjects, they were significantly lower than in the TP. Moreover, differences between patients with SP and healthy subjects were less pronounced in the dAAo and DAo than in patients with TP.

**Table 2:** Analysis of wall shear stress in patients with sinus prosthesis, tube prosthesis and healthy subjects

**WSS**_**avg**_	Pat SP		Pat TP		Healthy		p	p		
	Mean ± SD	95% CI	Mean ± SD	95% CI	Mean ± SD	95% CI	all	SP vs. TP	Healthy vs. SP	Healthy vs. TP
Bulb	0.85 ± 0.35	0.64; 1.06	0.96 ± 0.37	0.66; 1.25	0.92 ± 0.23	0.79; 1.05	0.79	0.53	0.56	0.80
Aao	0.57 ± 0.13	0.49; 0.65	0.82 ± 0.28	0.59; 1.05	0.60 ± 0.12	0.53; 0.66	**0.05**	**0.02**	0.61	**0.03**
dAAo	0.56 ± 0.13	0.48; 0.63	0.59 ± 0.16	0.46; 0.72	0.65 ± 0.15	0.57; 0.73	0.36	0.66	0.13	0.39
AA	0.52 ± 0.13	0.45; 0.60	0.50 ± 0.23	0.32; 0.68	0.64 ± 0.11	0.57; 0.70	0.06	0.82	**0.03**	0.10
DD	0.51 ± 0.16	0.42; 0.61	0.53 ± 0.13	0.42; 0.64	0.59 ± 0.15	0.51; 0.67	0.45	0.82	0.25	0.38
DAo	0.68 ± 0.23	0.55; 0.81	0.68 ± 0.18	0.53; 0.83	0.65 ± 0.12	0.59; 0.72	0.97	0.99	0.72	0.70
**max. WSS**_**seg**_	Pat SP		Pat TP		Healthy		**p**	**p**		
	Mean ± SD	95% CI	Mean ± SD	95% CI	Mean ± SD	95% CI	all	SP vs. TP	Healthy vs. SP	Healthy vs. TP
Bulb	1.19 ± 0.40	0.95; 1.43	1.73 ± 0.95	0.97; 2.49	1.17 ± 0.28	0.79; 1.05	0.58	0.11	0.88	0.07
Aao	0.97 ± 0.25	0.83 ± 1.12	1.83 ± 1.02	1.01; 2.26	0.82 ± 0.15	0.74; 0.90	**0.03**	**0.02**	0.61	**0.03**
dAAo	0.98 ± 0.28	0.81; 1.14	1.16 ± 0.40	0.84; 1.48	0.92 ± 0.16	0.82; 1.01	0.20	0.25	0.52	0.07
AA	0.80 ± 0.18	0.69; 0.90	0.80 ± 0.38	0.49; 1.10	0.84 ± 0.15	0.76; 0.93	0.08	0.99	0.53	0.71
DD	0.75 ± 0.29	0.58; 0.93	0.75 ± 0.19	0.60; 0.90	0.80 ± 0.15	0.72; 0.89	0.61	0.97	0.61	0.48
DAo	0.86 ± 0.27	0.71; 1.02	0.94 ± 0.19	0.79; 1.10	0.83 ± 0.12	0.76; 0.89	0.36	0.49	0.67	0.32
**WSS gradient**	Pat SP		Pat TP		Healthy		**p**	**p**		
	Mean ± SD	95% CI	Mean ± SD	95% CI	Mean ± SD	95% CI	all	SP vs. TP	Healthy vs. SP	Healthy vs. TP
Bulb	0.75 ± 0.25	0.61; 0.89	1.43 ± 0.90	0.70; 2.15	0.56 ± 0.12	0.49; 0.62	**< 0.01**	**0.03**	**0.02**	**< 0.01**
Aao	0.79 ± 0.23	0.65; 0.93	1.64 ± 1.04	0.80; 2.47	0.47 ± 0.18	0.37; 0.57	**< 0.01**	**0.02**	**< 0.01**	**< 0.01**
dAAo	0.76 ± 0.31	0.58; 0.94	1.01 ± 0.30	0.77; 1.25	0.47 ± 0.11	0.40; 0.53	**< 0.01**	0.10	**0.01**	**< 0.01**
AA	0.54 ± 0.18	0.43; 0.64	0.52 ± 0.35	0.23; 0.80	0.37 ± 0.13	0.30; 0.44	0.07	0.88	**0.02**	**0.02**
DD	0.56 ± 0.27	0.41; 0.72	0.57 ± 0.19	0.42; 0.72	0.45 ± 0.15	0.36; 0.53	0.42	0.97	0.20	0.13
DAo	0.37 ± 0.19	0.26; 0.49	0.5 ± 0.21	0.34; 0.67	0.31 ± 0.06	0.27; 0.35	**0.05**	0.18	0.29	**0.01**
**min. WSS**_**seg**_	Pat SP		Pat TP		Healthty		**p**	**p**		
	Mean ± SD	95% CI	Mean ± SD	95% CI	Mean ± SD	95% CI	all	SP vs. TP	Healthy vs. SP	Healthy vs. TP
Bulb	0.44 ± 0.36	0.23; 0.56	0.30 ± 0.16	0.18; 0.43	0.61 ± 0.21	0.49; 0.73	0.38	0.31	0.18	**< 0.01**
Aao	0.23 ± 0.10	0.17; 0.28	0.19 ± 0.17	0.05; 0.33	0.35 ± 0.18	0.24; 0.45	0.07	0.60	0.06	0.07
dAAo	0.22 ± 0.12	0.15; 0.29	0.15 ± 0.11	0.06; 0.24	0.45 ± 0.15	0.36; 0.54	**< 0.01**	0.25	**< 0.01**	**< 0.01**
AA	0.26 ± 0.15	0.18; 0.35	0.28 ± 0.23	0.09; 0.46	0.47 ± 0.11	0.41; 0.53	**0.01**	0.85	**< 0.01**	**0.02**
DD	0.24 ± 0.10	0.18; 0.30	0.22 ± 0.09	0.15; 0.29	0.36 ± 0.19	0.25; 0.46	0.09	0.65	0.08	0.07
DAo	0.49 ± 0.28	0.33; 0.65	0.44 ± 0.20	0.28; 0.60	0.52 ± 0.13	0.44; 0.59	0.37	0.66	0.77	0.30

Values for all investigated wall shear stress (WSS) parameters at six measurement contours in the aorta are given. Results of all three groups were tested for significance using Kruskal-Wallis-Test (all). In the column on the far right all three groups were tested in pairs, employing Man-Whitney-U-Test (SP vs. TP, SP vs. Healthy, TP vs. Healthy, respectively). Significant differences are marked in bold type

*Aortic bulb [bulb]; ascending aorta (AAo); distal ascending aorta (dAAo); aortic arch (AA); ductus diverticulum (DD); descending aorta (DAo); SP sinus prosthesis; TP tube prosthesis*

WSS_avg_ and max. WSS_seg_ were significantly different in the ascending aorta of patients with TP compared to patients with SP and healthy subjects. Both parameters were elevated in patients with TP compared to the other groups. There was a trend for increased values in the aortic bulb in these patients. Min.WSS_seg_ showed no significant differences between the two prostheses. In comparison to healthy subjects, the values in the aortic bulb, dAAo and AA were significantly lower in patients with both prostheses. With the exception of WSS_avg_ in the AA, all other parameters in the course of the vessel did not reach statistical significance.

Table 3 summarizes the results of quantitative flow, velocity and geometrical parameters. Maximum flow was significantly increased in both patient groups on all measurement sites, while maximum velocity only showed significantly higher values in the bulbus and the AAo. In both patient groups, the maximum vascular area was significantly increased in the native vessel distal to the prosthesis. Differences between both patient groups did not reach statistical significance.

**Table 3:** Analysis of quantitative parameters in patients with sinus prosthesis, tube prosthesis and healthy subjects

**Stroke volume**	Pat SP		Pat TP		Volunteer		p	p		
	Mean ± SD	95% CI	Mean ± SD	95% CI	Mean ± SD	95% CI	all	SP vs. TP	Vol vs. SP	Vol vs. TP
Bulb	99.4 ± 24.9	84.7; 114.1	90.3 ± 22.9	72; 108.6	84.8 ± 17	75.2; 94.5	0.22	0.43	0.11	0.55
AAo	93.1 ± 20.6	80.9; 105.2	78.9 ± 13.8	67.9; 90	76.4 ± 13	69; 83.8	0.15	0.11	0.03	0.68
dAAo	87.6 ± 21.7	74.8; 100.4	81.8 ± 13.7	70.8; 92.7	73.5 ± 16.5	64.1; 82.8	0.31	0.51	0.09	0.26
Arch	62.4 ± 21.3	49.8; 75	65.2 ± 13.5	54.3; 76	53.3 ± 9.9	47.7; 58.9	0.25	0.75	0.20	0.04
DD	58.3 ± 13.8	50.1; 66.5	54 ± 10.8	45.3; 62.7	48 ± 6.9	44.1; 51.9	0.17	0.48	**0.03**	0.14
DAo	60.1 ± 13.2	52.3; 67.9	52.1 ± 9.8	44.2; 59.9	48.8 ± 10.3	43; 54.7	0.28	0.16	**0.03**	0.49
**Max. flow**	Pat SP		Pat TP		Healthy		**p**	**p**		
	Mean ± SD	95% CI	Mean ± SD	95% CI	Mean ± SD	95% CI	all	SP vs. TP	Healthy vs. SP	Healthy vs. TP
Bulb	486 ± 114	418; 553	459 ± 53	417; 501	379 ± 99	324; 435	**0.05**	0.54	**0.03**	**0.05**
AAo	490 ± 92	436; 544	447 ± 39	415; 478	344 ± 75	302; 386	**< 0.01**	0.23	**< 0.01**	**< 0.01**
dAAo	433 ± 150	344; 521	443 ± 74	384; 503	318 ± 92	266; 370	**0.04**	0.85	**0.04**	**< 0.01**
AA	348 ± 73	305; 391	319 ± 44	284; 355	256 ± 66	218; 293	**< 0.01**	0.34	**< 0.01**	**< 0.01**
DD	304 ± 70	263; 346	304 ± 40	272; 337	230 ± 56	198; 262	**0.01**	0.99	**0.01**	**< 0.01**
DAo	285 ± 71	243; 327	284 ± 37	254; 314	210 ± 60	176; 243	**0.05**	0.97	**< 0.01**	**< 0.01**
**Max. velocity**	Pat SP		Pat TP		Healthy		**p**	**p**		
	Mean ± SD	95% CI	Mean ± SD	95% CI	Mean ± SD	95% CI	all	SP vs. TP	Healthy vs. SP	Healthy vs. TP
Bulb	161 ± 26	145; 176	186 ± 47	148; 224	133 ± 18	123; 144	**< 0.01**	0.15	**0.01**	**< 0.01**
AAo	148 ± 42	122; 173	148 ± 45	112; 183	97 ± 17	87; 106.4	**< 0.01**	0.99	**< 0.01**	**< 0.01**
dAAo	128 ± 35	107; 149	154 ± 44	119; 189	120 ± 79	75; 164	**0.03**	0.17	0.75	0.28
AA	104 ± 21	91; 116	108 ± 34	80; 135	93 ± 21	82; 105	0.28	0.75	0.26	0.27
DD	99 ± 31	80; 117	104 ± 48	66; 143	94 ± 18	84; 104	0.97	0.77	0.64	0.50
DAo	92 ± 26	76; 107	105 ± 26	84; 126	90 ± 15	82; 99	0.25	0.28	0.90	0.12
**Max. area**	Pat SP		Pat TP		Healthy		**p**	**p**		
	Mean ± SD	95% CI	Mean ± SD	95% CI	Mean ± SD	95% CI	all	SP vs. TP	Healthy vs. SP	Healthy vs. TP
Bulb	643 ± 233	505; 781	549 ± 175	409; 689	673 ± 150	589; 758	0.18	0.35	0.71	0.11
AAo	814 ± 215	687; 941	938 ± 245	741; 1134	736 ± 182	634; 839	0.13	0.26	0.36	**0.05**
dAAo	879 ± 300	702; 1057	964 ± 233	777; 1150	567 ± 145	485; 649	**< 0.01**	0.52	**< 0.01**	**< 0.01**
AA	738 ± 268	580; 896	729 ± 206	564; 894	462 ± 126	390; 534	**< 0.01**	0.94	**< 0.01**	**< 0.01**
DD	624 ± 291	452; 796	675 ± 151	554; 796	408 ± 102	351; 466	**< 0.01**	0.66	**0.02**	**< 0.01**
DAo	539 ± 277	375; 702	518 ± 131	413; 623	344 ± 72	303; 385	**< 0.01**	0.85	**0.03**	**< 0.01**

Values for all investigated parameters are given at six measurement contours in the aorta are given. Results of all three groups were tested for significance using Kruskal-Wallis-Test (all). In the column on the far right all three groups were tested in pairs (SP vs. TP, SP vs. Healthy, TP vs. Healthy, respectively) by means of Man-Whitney-U-Test. Significant differences are marked in bold type

### Secondary flow patterns and wall shear stress

Secondary flow patterns were detected in all VSARR patients and five healthy subjects. In spatial relation to the contours, secondary flow formations were found in the AAo in 23 study participants (11 SP; 6 TP; 6 Healthy), in the dAAo in 16 participants (8 SP; 8 TP), in the arch in 4 participants (1 SP; 3 TP) and at the DD in 8 participants (2 SP; 3 TP; 3 Healthy). No secondary flow patterns were detected in the bulb and the DAo in direct spatial relation to the contours. Of the total number of flow patterns analyzed, 42% were found in patients with SP, 45% in patients with conventional TP and 13% in healthy subjects. The flow patterns analyzed in the respective groups were classified into the grading system (Fig. 3; 1 = low-grade, 2 = moderate, 3 = high-grade) as follows: patients with SP 1 = 14% / 2 = 77% / 3 = 9%; patients with conventional TP 1 = 29% / 2 = 46% / 3 = 25%; healthy subjects 1 = 14% / 2 = 86% / 3 = 0%.

**Fig. 4:**
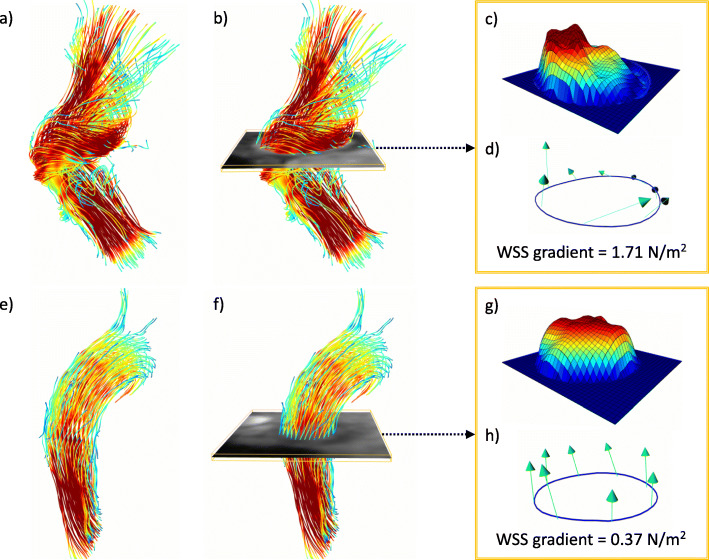
Distribution of WSS values and flow profiles in different flow patterns. a + b) depicts a patient with a conventional tube prosthesis developing a secondary helix in the ascending aorta. In the analysis plane shown in **b**), the local acceleration of flow at the outer curvature can be seen in the scalar map (**c**) resulting in large regional differences in wall shear stress (WSS) and a relatively high WSS gradient. In contrast, the healthy subject e) + f) shows a laminar, evenly distributed flow profile (**g**) and low WSS gradient (**h**). Flow was visualized in both subjects with 3D streamlines, color-coded with respect to flow velocity (V [cm/s])

**Fig. 5:**
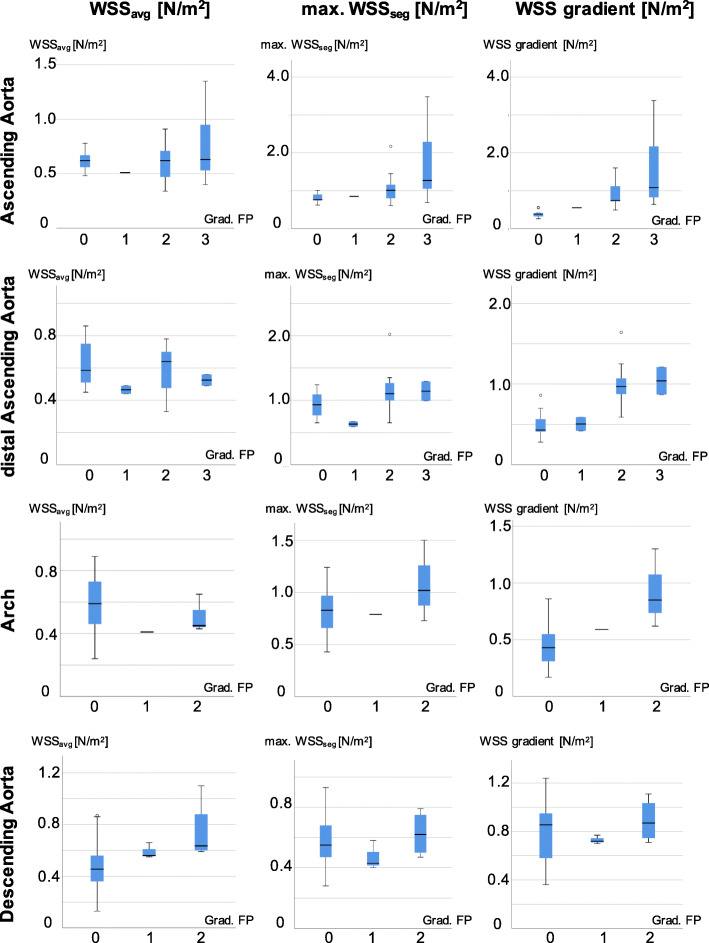
Boxplots for wall shear stress (WSS) parameters related to the formation of secondary flow patterns. WSS values at respective contours in the aorta are plotted against the grading of secondary flow patterns (Grad. FP) formed at the respective contour of the WSS measurement. In particular, the WSS gradient shows clearly increasing values with a higher grading of secondary flow patterns. While the influence of grade 1 flow patterns on all wall shear stress parameters is relatively small, the increase in WSS values in presence of grade 2 and grade 3 flow patterns is evident. The upper and lower borders of the box represent the upper and lower quartiles. The middle horizontal line represents the median, the upper and lower whiskers the maximum and minimum values of non-outliers. Outliers are marked as extra dots. Systematics of flow pattern grading is demonstrated in Fig. 3

Results of Spearman’s correlation coefficient can be found in Table 4. Significant correlations were found between the WSS gradient and the grading of secondary flow patterns at all measurement sites as well as for max. WSS_seg_ at the level of the AAo. Strong, but insignificant correlations were found for max. WSS_seg_ and WSS_avg_ in the AAo. The impact of secondary flow patterns on WSS is illustrated in Fig. 4. Box plots demonstrating the distribution of WSS values in relation to the grading of secondary flow patterns in the respective contours are shown in Fig. 5.

**Table 4:** Spearman’s correlation coefficients for wall shear stress parameters with secondary flow patterns

	**WSS gradient**	p	**max. WSS**_**seg**_	p	**WSS**_**avg**_	p
Bulb	–	–	–	–	–	–
AAo	0.77	**< 0.01**	0.52	**< 0.01**	0.84	0.65
dAAo	0.77	**< 0.01**	0.44	0.13	−0.18	0.32
Aao	0.52	**< 0.01**	0.20	0.27	−0.20	0.27
DD	0.51	**< 0.01**	0.13	0.49	0.58	0.75
DAo	–	–	–	–	–	–

Spearman’s correlation coefficients for wall shear stress (WSS) parameters at all six measurement contours in the aorta with grading of secondary flow patterns

Significant differences are marked in bold type

Results for the inter-reader comparison of WSS parameters and gradings of flow patterns yielded good to excellent results: ICC for WSS_avg_ was 0.72 [0.52–0.85], for max. WSS_seg_ 0.82 [0.69–0.91] and for the WSS gradient 0.76 [0.57–0.86], all *p* < 0.01. Cohen’s kappa coeffient comparing the grading of secondary flow patterns by both readers was κ = 0.86.

## Discussion

This study revealed that physiologically shaped SP seem to have a positive impact on aortic WSS compared to conventional TP. We introduced the WSS gradient as a new quantitative parameter to discriminate between different patient groups. Since the WSS gradient is strongly correlated not only with the expression but also with semi-quantitative grading of secondary flow patterns, it provides further evidence to link disturbed flow conditions to degenerative vessel wall changes.

WSS changes in patients were apparent not only at the level of the prosthesis but also in the course of the vessel, potentially inducing long-term pathological altering of the vessel architecture and increasing post-operative morbidity. Significant differences between both prostheses were found in and adjacent to the prostheses. Even if differences did not reach statistical significance, values in the SP were closer to the physiological values of healthy subjects than in the TP underlining the importance of aortic sinuses for hemodynamics. Comparing both prostheses to healthy subjects, significant differences in the AAo were only found for the TP but not the SP. Albeit comparison of different works is hampered by a lack of standardized protocols regarding the evaluation of WSS, the results in the aortic bulb and the AAo are in concordance with a previous study that assessed WSS in a prosthesis with neo-sinuses of a different vendor using a volumetric approach [16]. Arguably, Gaudino and colleagues also reported significant differences for WSS_avg_ and maximum WSS in the DAo, which was only partially reproducible in our study. Possible explanations can be found in manufacturer-specific characteristics of the prostheses, various WSS analysis protocols or the small number of patients in each study but remain speculative.

The WSS gradient is a newly introduced parameter, which has not been published before. The concept behind this parameter is to consider not only markedly increased WSS values but also strongly decreased values. While elevated WSS induces dilatation through increased activity of endothelial nitric oxide synthase and degradation of elastic fibers, reduced WSS is known to induce vessel constriction [23, 24]. Consequently, both, maxima and minima are of pathological relevance promoting endothelial dysfunction and resulting in opposing anatomical changes that have a stronger potential to impair hemodynamics than local WSS alterations [24]. However, WSS_avg_,max. WSS_seg_, and min WSS_seg_ may only partially portray these changes due to averaging and the exclusion of either peak or minimum values. Both shortcomings are overcome via the WSS gradient. However, one of the disadvantages of 4D flow CMR (e.g. compared to 2D flow CMR), due to the relatively low spatial and temporal resolution, and high signal-to-noise ratio, is an increased inaccuracy in the measurement of maximum and minimum values [25]. We have tried to reduce this drawback by not comparing the maximum and minimum values in individual voxels, but rather in eight regions orientated towards landmarks in the vessel’s geometry (Fig. 1), each grouped over several voxels. While the placement of the regions exhibits a potential source of error, good to excellent inter-reader agreement confirms the applicability of this approach.

Although there were marked differences between patients and healthy subjects when analyzing flow visually, established WSS parameters mostly exhibited insignificant tendencies between groups. However, the visually obvious differences in hemodynamics between groups could be quantified with the WSS gradient that was able to depict significant differences between both patient groups and healthy subjects with good inter-reader reproducibility. In both prostheses, the WSS gradient was increased compared to healthy subjects, especially in the dAAo and the AA. Still, measurements in the SP were closer to those of healthy subjects than in patients with TP. While established WSS parameters already indicated differences between the groups, in most cases these did not reach the significance level. This was only achieved by the WSS gradient, which seems to be more sensitive to differences of altered shear forces acting on the vessel wall than established parameters WSS_avg_ and max. WSS_seg_. The elevated WSS gradient coincides with an increased number of secondary flow patterns already discussed in a previous paper [8]. Secondary flow patterns are a result of regional accelerations and decelerations of flow and velocity as visualized in the scalar maps in Fig. 2. It seems straightforward that these altered hemodynamic conditions directly influence mechanical forces acting on the vessel wall and it has been hypothesized, that secondary flow patterns are not only a consequence of altered aortic geometry, but also induce its development [28]. The correlations between WSS parameters and a previously introduced semi-quantitative grading system of secondary flow patterns [8] provide evidence that an increased number of secondary flow patterns can potentially lead to postoperative aneurysm growth and therefore affect postoperative morbidity. As the WSS gradient was the parameter demonstrating the highest correlation, it may be suitable to quantify and follow-up the effects of disturbed blood flow on the vessel wall. Theoretically, the WSS gradient should be better at identifying vortices characterized by antegrade and retrograde flow in one cross section at the same time in contrast to helical flow. Unfortunately, the study collective was too small to generate the statistical power for such an analysis. Future studies could evaluate the possibility of discrimination between secondary helices and vortices including gradients of other WSS parameters such as axial and circumferential WSS. In both flow patterns there are differently pronounced flow accelerations, which potentially have different effects on the WSS and thus on the vessel wall architecture.

The results for hemodynamic and geometrical quantitative parameters are in concordance with a growing body of literature evaluating patients who underwent VSARR [11–14, 16, 29]. The significantly increased velocities at the level of the prosthesis most likely indicate the reduced compliance of both, the grafts’ material and the associated limited Windkessel function of the vessel [30]. This is also an explanatory approach for the significantly increased maximum flow in both prostheses during systole. Nonetheless, maximum velocities were lower in the SP, suggesting a limited reservoir function of the artificial sinus. Moreover, the SP allows for formation of vortices in the sinus [15]. Vortices, in general, have been discussed to preserve kinetic energy [17, 18] which may then be released later in the cardiac cycle, partly mimicking a Windkessel effect. Whether the increased maximum area of the aorta in patients is linked to post-prosthetic dilatation or the individual’s pre-existing vessel dilation remains hypothetical. Lower vessel area distal to the prostheses in SP compared to TP may be interpreted in favor of the SP, however, vessel areas in the descending aorta where slightly larger in patients with SP. Since patients were not examined before surgery, it is possible that any differences existed beforehand and were not related to the type of prosthesis.

Overall, these findings add to the ongoing discussion about the role of the aortic sinuses by offering quantitative proof of improved flow conditions in the anatomically pre-shaped SP. Although no differences in the clinical outcome have been reported so far [31], Guzzardi et al. and Bollache et al. have impressively demonstrated the thinning of elastic fibers at regions of altered WSS in the aorta [23, 24]. Hence, an effect on the architecture of the aortic vessel wall is highly probable, but may only become evident over the course of several decades. As VSARR is increasingly used in younger patients to maintain function of the native aortic valve [32, 33], this may promote the manifestation of late post-operative morbidity.

Despite the positive influence on WSS and hemodynamics as described above, several quantitative parameters still show significant differences between patients with SP and healthy subjects. Besides the limited compliance of graft material, the SP is primarily designed to imitate the geometry of the aortic bulb to allow for smooth closure of the aortic valve and perfusion of the coronaries [34, 35]. In the course of the prosthesis and the distal aorta, no measures are taken to improve aortic geometry, resulting in kinking of the prosthesis or at the distal anastomosis as well as post-prosthetic dilatation in both patient groups [8]. To our experience, the post-procedural geometry of the AA substantially affects flow conditions in the DAo [36]. As described in a previous paper, the aortic geometry of our patients, regardless of the prosthesis, differed considerably from healthy subjects [8]. The two patients added to the TP group in this paper also exhibited a more angulated “cubic” aortic anatomy compared to the “round” aortic arch of healthy subjects. This provides a possible explanation for the lack of differences in WSS and other quantitative parameters in the distal aorta. An approach to overcome this limitation is the implantation of so-called 90° prostheses, which attempt to emulate the natural shape of the AAo. Additional analyses, including these prostheses as well as developing methods to quantify the geometry of the aorta, may further help our understanding of post-procedural hemodynamics after VSARR and improve surgical strategies.

### Limitations

Potential limitations of the study can be seen in the limited size of the patient population. Although of interest, the small number of subjects prevents a correlation analysis of secondary flow patterns and the WSS gradient for each separate group due to the limited statistical power. Factors influencing hemodynamics are manifold and assembling a homogenous patient collective remains a challenge. Since surgery was often performed in an emergency setting (e.g. AAo dissection) with immediate need for action, it was not possible to perform preoperative measurements. As the primary objective of the study was to evaluate the ability of the two different prostheses to achieve close to physiological flow conditions post-operatively, the comparison with an age-matched collective embodying these characteristics seems scientifically sound. As 4D flow CMR was performed after surgery on a voluntary basis, pre-selection bias in patients cannot be excluded. Beside the aorta’s characteristics, morphology of the aortic valve may be a factor directly affecting flow conditions [24, 37]; respective analysis would be of great interest but were beyond the scope of this work.

The general limitations of 4D Flow CMR are well known and have been discussed extensively [25]. As investigating hemodynamics post-VSARR with 4D flow CMR is a novel, emerging field, the clinical significance of 4D flow parameters is unclear; the actual effects on the vessel wall remain in part theoretical and larger longitudinal studies are necessary to investigate the clinical correlation of the collected parameters with post-operative morbidity. With 4D Flow CMR being an evolving method with rapid advances in sequence and post processing techniques, there exists no standardized protocol or cut-off values to evaluate wall shear stress. This hampers comparisons of populations investigated in other studies.

## Conclusion

Physiologically shaped SP seem to have a positive impact on the mechanical forces affecting the aortic wall compared to straight TP in patients after VSARR. Nonetheless, significant differences in WSS, hemodynamic, and geometrical parameters are still present and call for further optimization of surgical techniques and prostheses characteristics. These may include more compliant material and individually tailored grafts, adaptive to the native aortic anatomy.

It should be noted that although established WSS parameters revealed tendentious differences between patients, these did not reach the significant level. We present the novel parameter “WSS gradient”, which seems to be more sensitive to changes in WSS than established WSS parameters. Moreover, the WSS gradient correlates with the occurrence and strength of secondary flow patterns. It therefore links secondary flow patterns to pathological, degenerative vessel wall changes potentially affecting post-procedural morbidity. However, while the effect of WSS on the vessel wall is well validated on a histopathological level and different pilot-studies link altered WSS to various pathologies, long-term follow-up studies evaluating the clinical impact of the WSS changes in different patient collectives are warranted.

## Data Availability

The datasets used and/or analyzed during the current study are available from the corresponding author on reasonable request.

## Abbreviations

4D Flow CMR: Time-resolved 3-dimensional flow cardiovascular magnetic resonance
AA: Aortic arch
AAo: Ascending aorta
CMR: Cardiovascular magnetic resonance
dAAo: Distal ascending aorta
DAo: Descending aorta
DD: Ductus diverticulum
ECG: Electrocardiogram
SENSE: Sensitivity encoding
SP: Sinus prosthesis
TP: Tube prosthesis
VENC: Velocity-encoding
VSARR: Valve sparing aortic root replacement
WSS: Wall shear stress
WSS_avg_: Averaged wall shear stress
WSS_seg_: Maximum segmental wall shear stress
